# Lateral Crural Mid-Down Flap Technique in Primary Rhinoplasty

**DOI:** 10.1007/s00266-025-04896-8

**Published:** 2025-05-20

**Authors:** Sina Kaderi, Can Ekinci, Yakup Karabağlı

**Affiliations:** 1 Private Practice, Bostancı. Bağdat Av. Çınarlı St. No: 10 Apt:7, Kadıköy/İstanbul, 34744 Turkey; 2 Department of Plastic, Reconstructive and Aesthetic Surgery, Eskişehir Osmangazi University School of Medicine, Eskişehir, Turkey

**Keywords:** Bulbous, Flap, Lateral crus, Malposition, Nasal tip, Rhinoplasty

## Abstract

**Background:**

Nasal tip surgery, especially the correction of deformities of the lateral crura, is perhaps one of the most difficult areas of rhinoplasty, and many different techniques have been described in the literature.

**Objectives:**

The authors describe a new flap technique for the correction of excessive convexity of lateral crura causing boxy, bulbous, and parentheses tip deformities.

**Methods:**

Thirty patients were randomly divided into two groups with equal numbers of male and female patients in each group. In half of the patients, lateral crural correction was performed with the cephalic excision (CE) method, while in the other half, it was performed with the lateral crural mid-down flap (LCMF) technique described by the authors. SCHNOS (standardized cosmetic and health nose outcomes questionnaire), nasal pinching questionnaire, and VAS (visual analog scale) were used to evaluate the results within and between groups.

**Results:**

While significant improvement was observed in both techniques according to SCHNOS and VAS compared to preoperative evaluation, no significant difference was observed between the two groups. On the other hand, in the nasal pinching questionnaire, which includes more specific questions about the nasal tip aesthetics and nasal valve function, better postoperative results were obtained in the LCMF technique, while deterioration was observed in the CE technique according to preoperative findings.

**Conclusion:**

The LCMF technique can be considered as an easy and suitable technique for the correction of lateral crura, especially in patients with excessively convex lateral crura with a bulbous nasal tip.

**Level of Evidence IV:**

This journal requires that authors assign a level of evidence to each article. For a full description of these Evidence-Based Medicine ratings, please refer to the Table of Contents or the online Instructions to Authors www.springer.com/00266.

**Supplementary Information:**

The online version contains supplementary material available at 10.1007/s00266-025-04896-8.

## Introduction

Nasal tip surgery is perhaps the area in rhinoplasty where there are the greatest divergences among surgeons and where many different surgical techniques are developed [1]. For this purpose, methods using cartilage reshaping, different types of grafts, and different suture techniques have been developed and used in the literature [2]. Which of these techniques will be preferred should be decided according to the preoperative evaluation of the patient and patient-based technique selections should be made.

One of the areas where surgeons have the most difficulty in tip-plasty surgeries is the correction of malpositioned or poor shaped lateral crura, and it is not easy to correct these deformities [3]. Excessive convexity of lateral crura can cause boxy, bulbous, and parentheses tip deformities [4]. Many surgical techniques have been proposed for the correction of deformities in the lateral crura, but there is still no gold standard surgical technique and new techniques are also suggested [5, 6].

These techniques can be generally described as reshaping the lateral crura with sutures [7, 8], lateral crural cephalic trim [9, 10], correcting the concavity by reversing the lateral crura [11], using alar rim grafts [12], using turn-in flaps [6], and using overlay [13] or underlay [14] grafts as normal or extended [3]. While all these techniques have advantages over each other, they also have shortcomings [2]. Among these techniques, the technique that can be said to provide significant stabilization is the use of lateral crural strut grafts [2, 5]. However, this technique generally requires the use of cartilage grafts obtained from the septum or from a secondary surgical site, such as the costa or concha, and prolongs the surgery [3, 5]. Therefore, in secondary rhinoplasty cases, it may not be possible to perform this technique or it may be necessary to take a graft from the secondary surgical site. Considering all these limitations and the prolongation of surgery, cephalic excision is the more preferred and basic method, especially in patients with boxy and bulbous tips with excessively concave lateral crura [9, 10, 15].

In this article, we will present our technique for correcting the excessive convexity in the lateral crus by using part of the lateral crus itself as a mid-down flap without using a graft from a different donor area. The results of this technique are compared with the results of the cephalic excision technique. In the lateral crural mid-down flap (LCMF) technique, since the support is provided from the central region, it is more effective in correcting the excessive convexity of the lateral crura and prevents it from causing concavity of the lateral crura. In addition, since a flap is used instead of a graft, problems such as displacement of the graft are not encountered.

## Methods

This study was approved by the Institutional Ethical Review Board of a University Medical School (07.01.2020, Decision Number: 08). The study was designed in a retrospective manner, and informed consents were obtained from all the patients included in the study. Additional written informed consents for patient information and images to be published were provided by the patients for whom identifying information is included in this article.

Medical and personal records of thirty patients operated between November 2017 and September 2018 were retrospectively assessed. Half of the patients were randomly selected and operated on with the lateral crural mid-down flap (LCMF) technique, while the cephalic excision (CE) technique was used as a control technique in the remaining fifteen patients.

Inclusion criteria were primary rhinoplasty patients with bulbous tip having excessive convexity of lateral crura, minimum 12-month follow-up, patients being over 18 years of age, pre- and postoperative photographic documentation, and completion of the SCHNOS (standardized cosmesis and health nasal outcomes survey) [16] (Table 1), nasal pinching questionnaire (Table 2), and VAS (visual analog scale) [17] (Table 3) assessed by the patients and 4 independent experts. Secondary rhinoplasty cases, patients with comorbidities, patients with serious perioperative respiratory problems, and patients with severe dorsal hump or septum deviation were not included in the study.

**Table 1:** Standardized cosmesis and health nasal outcomes survey (SCHNOS) [16]

	No problem					Extreme problem
1. Having a blocked or obstructed nose	0	1	2	3	4	5
2. Getting air through my nose during exercise	0	1	2	3	4	5
3. Having a congested nose	0	1	2	3	4	5
4. Breathing through my nose during sleep	0	1	2	3	4	5
5. Decreased mood and self-esteem due to my nose	0	1	2	3	4	5
6. The shape of my nasal tip	0	1	2	3	4	5
7. The straightness of my nose	0	1	2	3	4	5
8. The shape of my nose from the side	0	1	2	3	4	5
9. How well my nose suits my face	0	1	2	3	4	5
10. The overall symmetry of my nose	0	1	2	3	4	5

**Table 2:** Nasal pinching questionnaire

	Not at all					Advanced
Is there a pinched nasal tip present?	0	1	2	3	4	5
Is there a nasal valve collapse during normal inspiration?	0	1	2	3	4	5
Is there a nasal valve collapse during forced inspiration?	0	1	2	3	4	5
What is the level of relief with Cottle maneuver?	0	1	2	3	4	5

**Table 3:** Visual analog scale (VAS) [17]

	Very bad									Very good
Evaluation by the patient	1	2	3	4	5	6	7	8	9	10
Evaluation by the first expert	1	2	3	4	5	6	7	8	9	10
Evaluation by the second expert	1	2	3	4	5	6	7	8	9	10
Evaluation by the third expert	1	2	3	4	5	6	7	8	9	10
Evaluation by the fourth expert	1	2	3	4	5	6	7	8	9	10

The patients' preoperative complaints and surgical desires, intraoperative findings, postoperative complications such as infection and epistaxis, satisfaction with the shape of the nasal tip, and desire for revision surgery were evaluated in detail.

### Surgical Techniques

All patients underwent surgery under standard general anesthesia conditions and were operated on using the open rhinoplasty technique. While the lateral crural mid-down flap (LCMF) technique was used in half of the patients, the remaining fifteen patients constituted the control group using the cephalic excision (CE) technique.

Nasal dorsum, lateral nasal walls, tip, and septum were infiltrated with 1% lidocaine hydrochloride and 1:100,000 epinephrine. After the marginal incisions, a transcolumellar V-shaped incision was made and these incisions were joined. Upper lateral cartilages (ULCs) were exposed through subperichondrial dissections while during dissection of ULCs, care was taken to preserve the Scroll area and ligament. The Pitanguy ligament was cut but marked, preserved, and reconstructed at the end of the surgery. The dissection was advanced subperiostally in the nasal bone area.

Septal exposure was started initially from the anterior septal angle, and bilateral mucoperichondrial flaps were created under the subperichondrial plane. A great care was taken not to harm mucoperichondrial flaps which could potentially cause cicatricial narrowing of the internal nasal valve. To avoid damaging the ULCs during excision of the dorsal cartilaginous and bony hump, the ULCs were separated from the septum. Cartilaginous and bony hump was reduced according to the component dorsal hump reduction technique [18].

Routine medial and lateral osteotomies were performed if required. The open roof deformity was reconstructed with spreader flaps [19] or ULC fold-in flaps [20]. Lateral crural shaping was then performed, and the LCMF technique was applied to randomly selected fifteen patients, while the CE method was applied to the other half. The surgery was concluded by performing tip-plasty at the last stage of the surgery using routine techniques.

**Fig. 1:**
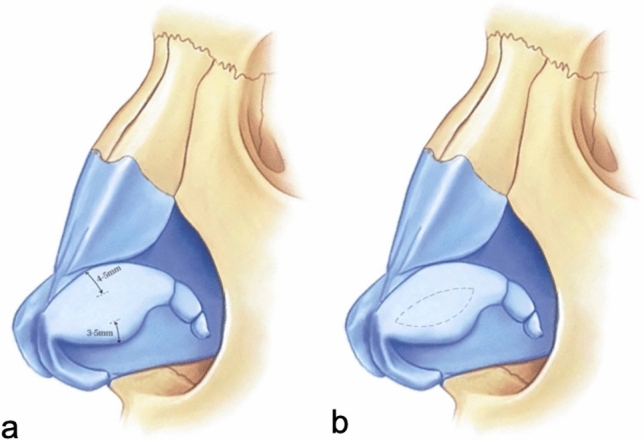
**a** Demonstration of the markings made at the cephalic and caudal border of the lateral crus. **b** Demonstration of marking the incision to be made

**Fig. 2:**
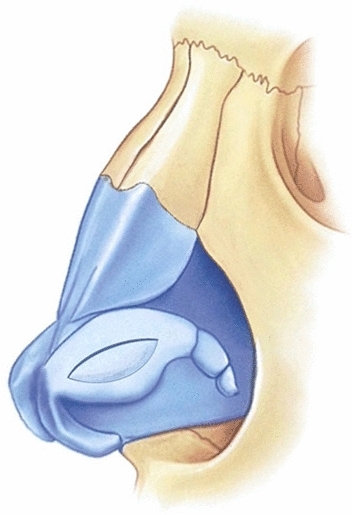
Demonstration of the lateral crural mid-down flap (LCMF) formed after incision

**Fig. 3:**
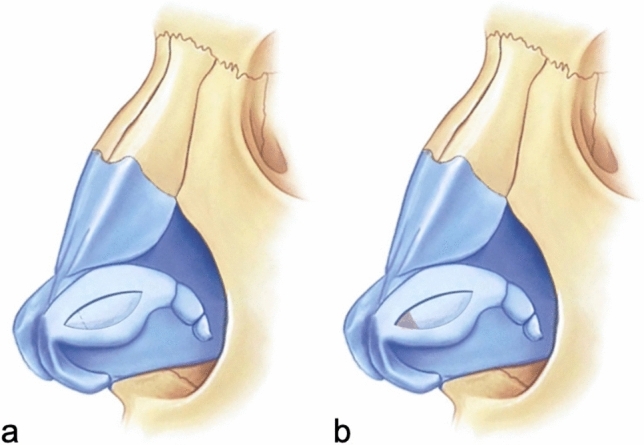
**a** Demonstration of marking the cartilage to be excised from the anterior third of the LCMF flap. **b** Demonstration of the flap formed after excision of the anterior third cartilage of the LCMF flap

To explain the LCMF technique in detail, firstly the lateral crura which were exposed by subperichondrial dissection are marked with a caliper. Two markings are made on the lateral crura, 4–6 mm from the cephalic border and 3–5 mm from the caudal border. Since these two markings determine the width of the lateral crura that will remain at the end of the procedure, the total width of these two markings is planned to be 7–8 mm in women and 9–11 mm in men (Fig. 1). In order to prevent mucosal damage and decrease bleeding, local infiltration is performed in the area between the cartilage and mucosa before incision. After local infiltration, an incision is made according to the markings (Fig. 2). In order not to complicate the placement of steal sutures during the tip-plasty stage, the anterior 1/3 of the cartilage of the flap, which is formed after the incisions and will form our LCM flap, is excised (Fig. 3).

**Fig. 4:**
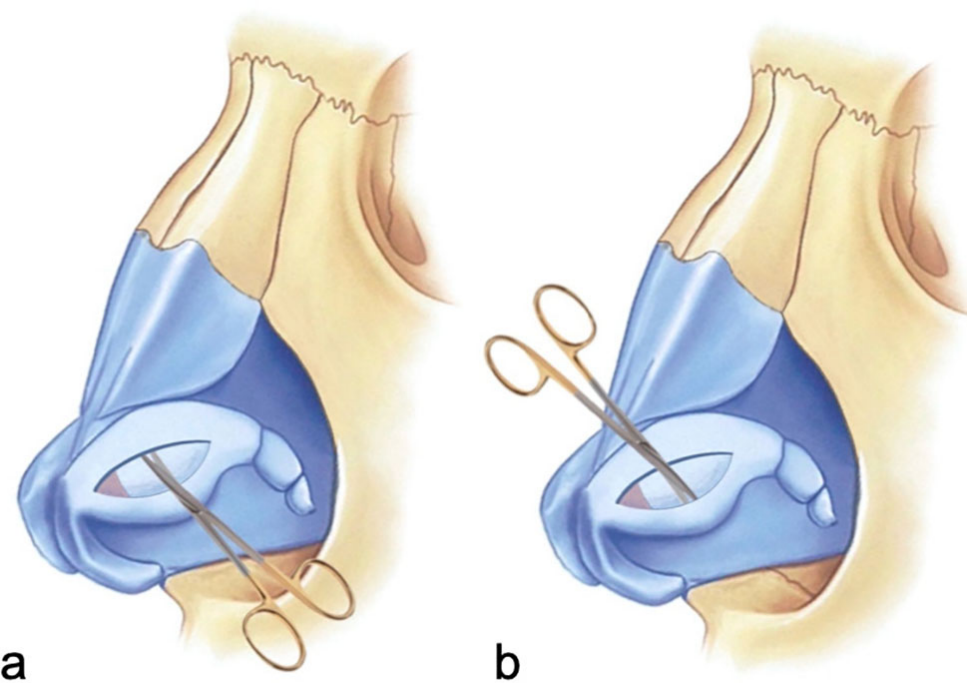
**a** Demonstration of pocket creation on the cephalic side. **b** Demonstration of pocket creation on the caudal side

**Fig. 5:**
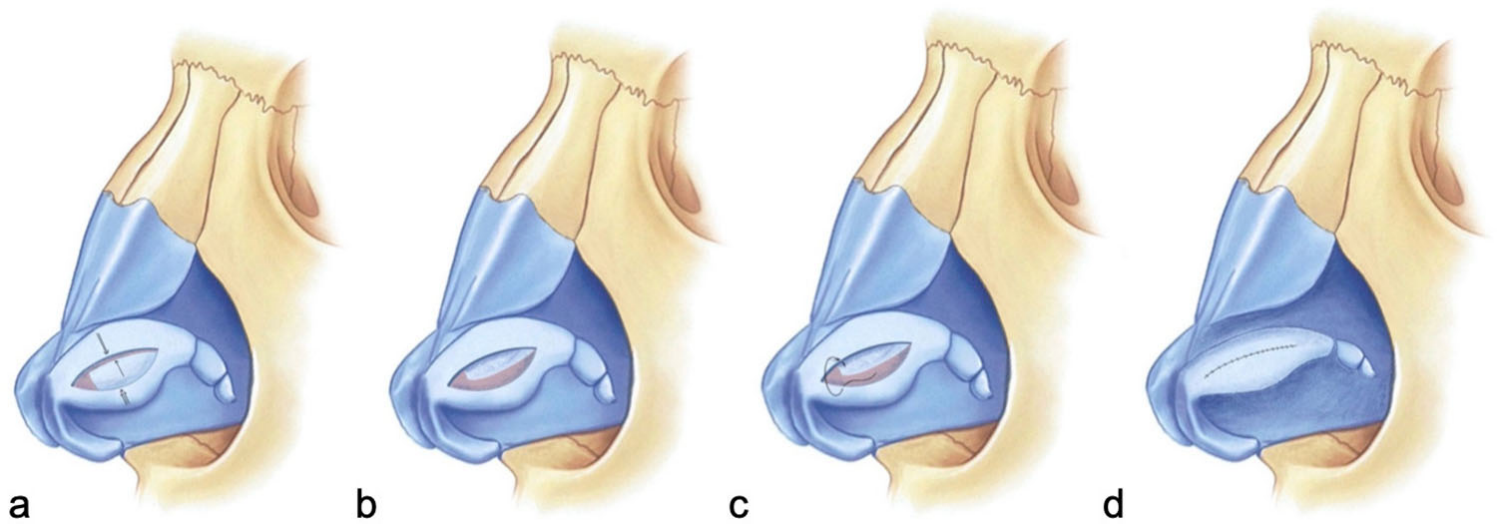
**a** Demonstration of LCMF placement in the pocket in the cephalic direction. **b** Demonstration of pulling of the caudal pocket over the LCMF. **c** Demonstration of suturing the cartilages forming the pockets to each other with 5/0 PDS to cover the LCMF. **d** Demonstration that the cephalic and caudal flaps completely cover the LCMF after suturing

**Fig. 6:**
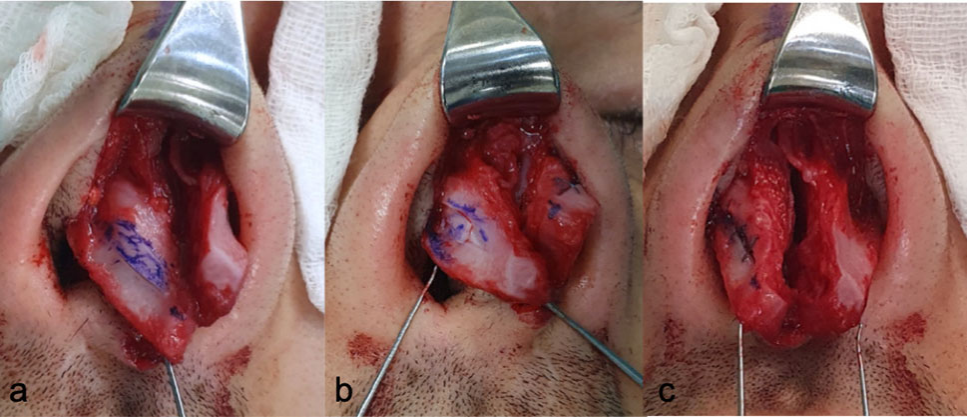
Intraoperative photos **a** Demonstration of the lateral crural mid-down flap (LCMF) formed after incision. **b** Demonstration of the incision of the cartilage to be excised from the anterior 1/3 of the LCMF. **c** Demonstration of the final appearance of the cephalic and caudal flaps after suturing, showing complete closure of the LCMF

The pocket in which the LCMF will be placed is prepared by dissections between the cartilage and mucosa in the cephalic and caudal areas previously marked on the lateral crus (Fig. 4). If the LCMF is larger than the created pockets or if bulging occurs when placed inside the pockets, then crescent-shaped strips are excised from both sides of the cartilage of the flap to make it compatible with the pockets. After making sure that it fits into the pockets, the LCMF is placed inside the pocket in the cephalic direction, and then, the caudal pocket is pulled over it and the cartilages forming both pockets are sutured with 5/0 PDS to cover the LCMF (Fig. 5). With this technique, while the widths of the lateral crura to be created are determined with the caudal and cephalic markings made at the beginning, support will be applied from the center of the new lateral crura with LCMF to correct the concavity (Fig. 6).

### Statistical Analysis

IBM SPSS (IBM Corp., Armonk, NY, USA) for Windows 21 was used in data analysis. Crosstabs were prepared according to the preoperative and postoperative values obtained according to SCHNOS, nasal pinching questionnaire, and VAS, and the marginal homogeneity test was used in the analysis. For the presentation of the data, number (%) values were used. The significance level was set at *P* < 0.05.

## Results

Twenty-four (80%) of the patients were female and six (20%) were male, and their ages ranged from 18 to 48 years (mean 28.68 years). Patients were randomly assigned to the LCMF and CE groups with equal numbers of female and male patients, with 12 (80%) female and 3 (20%) male patients in each group. The age distribution in the LCMF group ranged from 18 to 48 years (mean 29.6 years), while the age range in the CE group was from 19 to 38 years (mean 27.4 years). There was no significant difference between the groups in terms of age (*p*=0.547) (Supplementary Table 1).

Preoperative and postoperative results obtained with LCMF and CE techniques were evaluated within and between groups using SCHNOS (standardized cosmetic and health nose outcomes questionnaire), nasal pinching questionnaire, and VAS (visual analog scale), and statistical analyses were performed to compare the results. According to SCHNOS, there was a significant postoperative improvement within the groups compared to the preoperative results (*p*=<0.001), while no statistically significant difference was found between the two techniques (*p*=0.109) (Table 4).

**Table 4:** Statistical results of the SCHNOS (standardized cosmesis and health nasal outcomes survey) within and between groups

SCHNOS	Preoperative (mean)	Postoperative (mean)	*p*-Value	*p*-Value (comparison between groups)
Lateral crural mid-down flap (LCMF)	67.6	5.2	<0.001	0.109
Cephalic excision (CE)	62.9	14.13	<0.001	

When evaluated according to the nasal pinching questionnaire, a significant postoperative difference was observed between the groups compared to the preoperative results (*p*=<0.001). However, this difference was seen as improvement in the LCMF group and disimprovement in the CE group. Furthermore, this time the difference between the groups was also found to be significant (*p*=<0.001) (Table 5).

**Table 5:** Statistical results of the nasal pinching questionnaire within and between groups

Nasal pinching questionnaire	Preoperative (mean)	Postoperative (mean)	*p*-Value	*p*-Value (comparison between groups)
Lateral crural mid-down flap (LCMF)	35.0	11.0	<0.001	<0.001
Cephalic excision (CE)	29.7	40.0	0.030	

When evaluated by the patient and 4 independent experts according to VAS, a significant postoperative improvement was found in both groups (*p*=<0.001). When the two groups were compared with each other, it was seen that the difference was not significant (*p*=0.183) (Table 6). Numerical data of the tests used in the study are given in Supplementary Table 2.

**Table 6:** Statistical results of the visual analog scale (VAS) within and between groups

VAS	Preoperative (mean)	Postoperative (mean)	*p*-Value	*p*-Value (comparison between groups)
Lateral crural mid-down flap (LCMF)	39.6	86.0	<0.001	0.183
Cephalic excision (CE)	40.5	81.7	<0.001	

**Fig. 7:**
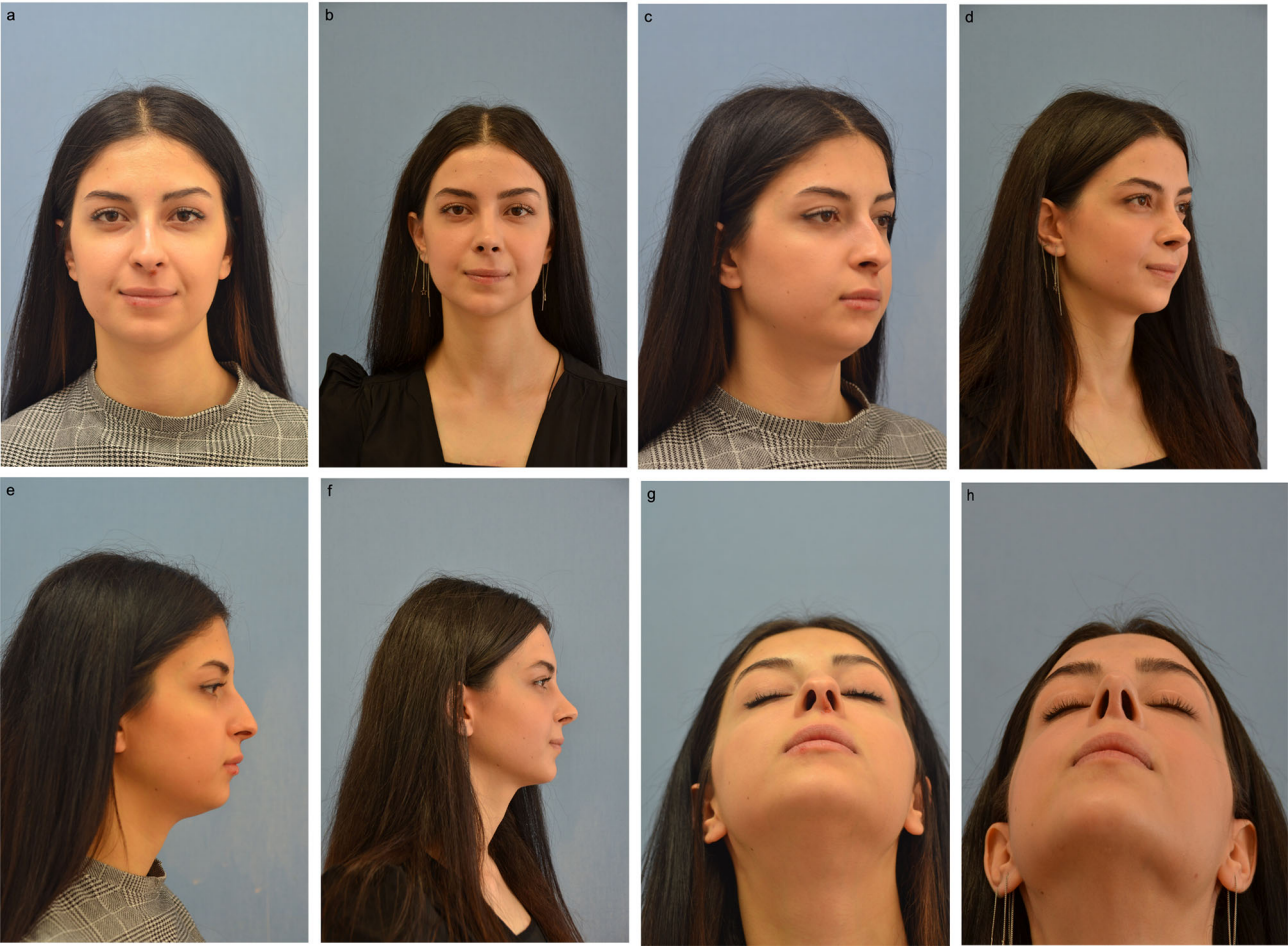
(**a**, **c**, **e**, **g**) Preoperative images of 22-year-old female presenting with high radix, dorsal hump and droopy and bulbous tip with excessively concave lateral crura. (**b**, **d**, **f**, **h**) Postoperative images one year after the primary rhinoplasty with lateral crural mid-down flap (LCMF) technique. Nasal tip rotation and projection were increased, and weak lateral crura were corrected with LCMF technique

**Fig. 8:**
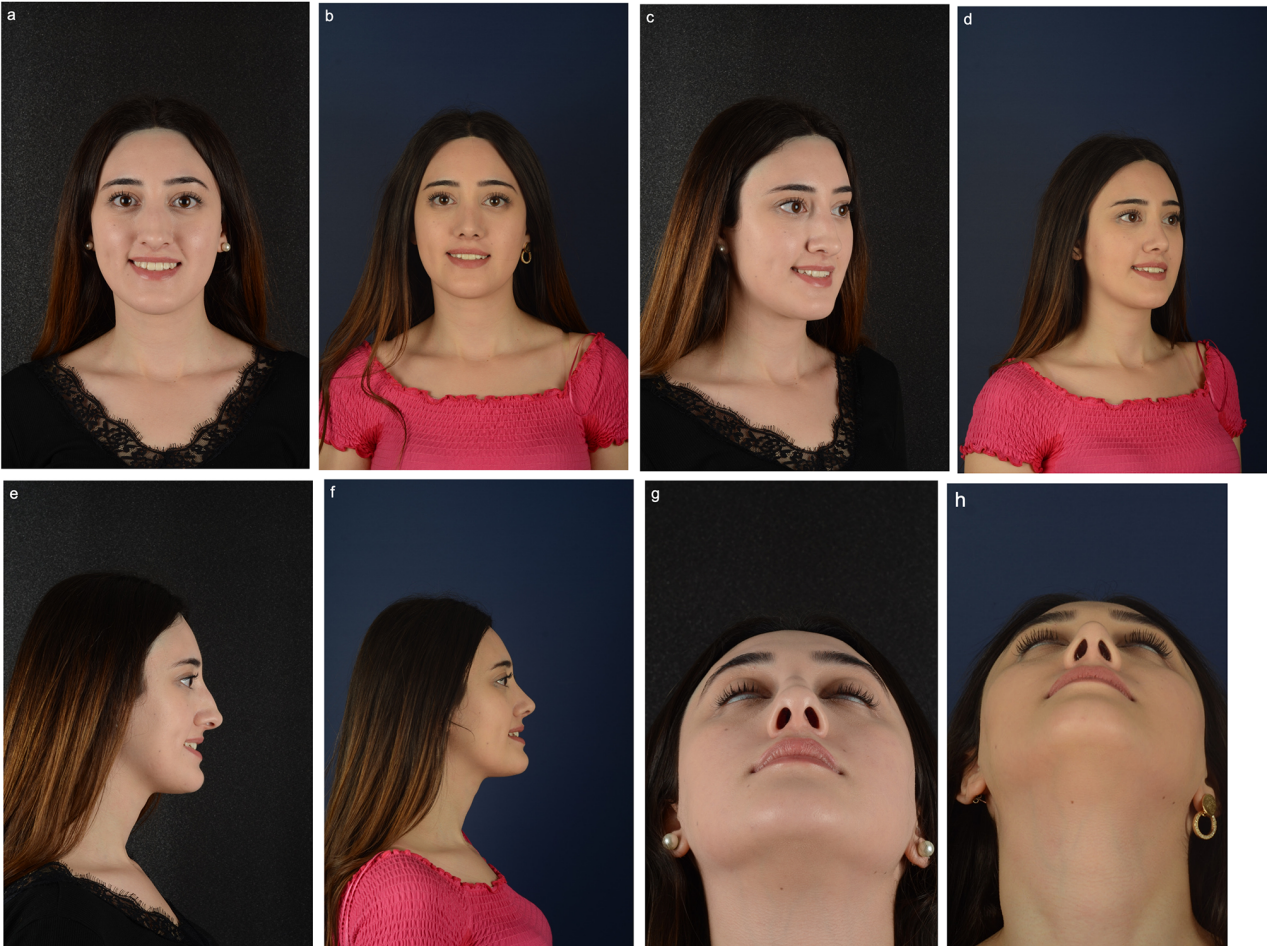
(**a**, **c**, **e**, **g**) Preoperative images of 19-year-old female with dorsal nasal hump and bulbous nasal tip with weak lateral cartilages. (**b**, **d**, **f**, **h**) Postoperative images three months after the primary rhinoplasty with lateral crural mid-down flap (LCMF) technique. Nasal hump was removed; bulbous nasal tip and weak lateral cartilages are corrected with LCMF technique

Clinical results are shown in Figs. 7 and 8.

## Discussion

Perhaps the most challenging area for surgeons in the field of rhinoplasty is nasal tip surgery, where many different techniques can be performed [1]. Within nasal tip surgery, it is quite difficult to correct weak and deformed lateral crura, and for this purpose different excision [9, 10], suturing [7, 8], grafting [12], or different flap [1, 6] techniques have been developed [2]. Among all these techniques, which technique to use should be determined for each patient and unfortunately there is no “gold standard” technique that is preferred over others [2].

Among the techniques used to correct deformities in the lateral crura, perhaps the technique that provides the most stabilization is the use of lateral crural strut grafts [2, 5]. However, the need for cartilage from a different donor area and the prolongation of surgery constitute disadvantages [21, 22]. There may also be drawbacks such as graft displacement or palpation of the graft under the alar skin [22]. Therefore, cephalic excision (CE) technique, which is a relatively easier and faster technique, is usually preferred, especially for the bulbous nasal tips where the concavity of the lateral crura is present [9, 10, 15].

In the described lateral crural mid-down flap (LCMF) technique, the width of the wide and concave lateral crura is reduced similarly as in the CE technique, but instead of removing the cartilage, it is placed as a flap to support the lateral crura from the center. Since the cartilage support is provided as a flap, negative effects such as graft displacement or palpation of the graft under the skin [22] are not observed. On the other hand, correction of the concavity of the lateral crura is achieved with strong support from the center. In addition, as stated in the studies of Janeke and Wright, the Scroll ligament is one of the four main fulcrums that provide nasal tip support [23], and since the Scroll area is preserved in the LCMF technique, nasal tip support is much better than in the CE technique. Although a similar technique to LCMF was described by Özmen et al. under the name of “Sliding alar cartilage flap” [24], LCMF technique provides more advantages in terms of nasal tip support due to the preservation of the Scroll area and ligament. Since the LCMF technique preserves the relationship between the upper and lower lateral cartilages, complications such as pinched tip nose, loss of tip projection, nasal tip drooping, and many others are seen less common [25].

When performing the LCMF technique, approximately 1/3 of the anterior flap cartilage should be removed, as in the “modified sliding alar cartilage flap” technique [26], so as not to interfere with the steal sutures during the tip-plasty stage, and thus, the polygons at the nasal tip [27] should be created aesthetically. Another important point is that if the flap is much larger than the pocket to be placed in it, it will create a bulge, so the flap should be reduced as necessary. Otherwise, the shape of the lateral crura to be created will be distorted and the ideal lateral crural resting angle [27] will not be achieved.

When the results were examined, significant improvement was observed in both techniques compared to the preoperative evaluation according to the SCHNOS, a 10-question questionnaire that evaluates aesthetic outcome and functionality. When the two techniques were compared with each other according to SCHNOS, no significant difference was found. On the other hand, when the nasal pinching questionnaire, which similarly evaluates both aesthetic results and functionality but has more specific questions regarding the nasal tip and nasal valve, was examined, it was seen that the LCMF technique was significantly better. In fact, it was observed that worse outcomes were obtained with the CE technique compared to the preoperative evaluation. On the other hand, when evaluated according to the VAS performed by the patient and 4 independent experts, better results were obtained in both techniques compared to the preoperative evaluation and both techniques were found to have significantly better aesthetic results. From these results and the fact that the deterioration in the nasal pinching questionnaire responses was mostly due to the nasal valve function and the relaxation of breathing with the Cottle maneuver, it can be said that the aesthetic results were similar in both techniques, but the functional results were significantly better in the LCMF technique. The reason why functional results are better in LCMF can be thought to be due to the fact that LCMF prevents nasal valve collapse by preserving the Scroll area and ligaments [28].

If the limitations of this study are evaluated, they can be listed as: The small number of patients included in the study and the technique compared was CE, which is an excision technique. Randomized studies using different graft or flap techniques with larger patient numbers and more objective functional evaluation tests may shed light on the comparison of the LCMF technique with other techniques available in the literature.

## Conclusion

In the LCMF technique, the connection between the upper and lower lateral cartilages, namely the Scroll area and ligament, is preserved; thus, the nasal valve is protected, which provides superior aesthetic results as well as better functional results, especially when compared to the CE technique. Advantages of the LCMF technique include that it does not require a cartilage graft from a secondary area and that it does not have complications such as graft displacement or palpation of the graft under the skin.

## Supplementary Information

Below is the link to the electronic supplementary material.

Supplementary file1 (DOCX 15 KB)

Supplementary file2 (DOCX 14 KB)
