# Supplementary material for: Lateral Crural Mid-Down Flap Technique in Primary Rhinoplasty
Source: Aesthetic Plast Surg. 2025 May 20;49(19):5431–9. doi: 10.1007/s00266-025-04896-8 (PMC12594689; doi:10.1007/s00266-025-04896-8)
Supplement: Supplementary file 1 — Supplementary file1 (DOCX 15 KB) [file 266_2025_4896_MOESM1_ESM.docx]

Supplementary Table 1.: General patient features

| Patient No. | Age | Gender | Follow-Up Duration (Month) | Technique |
| --- | --- | --- | --- | --- |
| 1 | 20 | F | 18 | LCMF |
| 2 | 42 | F | 18 | LCMF |
| 3 | 29 | F | 17 | LCMF |
| 4 | 32 | M | 17 | LCMF |
| 5 | 27 | F | 17 | LCMF |
| 6 | 18 | F | 16 | LCMF |
| 7 | 26 | M | 16 | LCMF |
| 8 | 19 | F | 15 | LCMF |
| 9 | 36 | F | 15 | LCMF |
| 10 | 45 | F | 14 | LCMF |
| 11 | 33 | M | 13 | LCMF |
| 12 | 48 | F | 13 | LCMF |
| 13 | 21 | F | 13 | LCMF |
| 14 | 30 | F | 12 | LCMF |
| 15 | 18 | F | 12 | LCMF |
| 16 | 20 | F | 19 | CE |
| 17 | 29 | M | 18 | CE |
| 18 | 32 | F | 18 | CE |
| 19 | 30 | F | 17 | CE |
| 20 | 19 | F | 17 | CE |
| 21 | 31 | F | 17 | CE |
| 22 | 36 | F | 16 | CE |
| 23 | 25 | F | 15 | CE |
| 24 | 19 | M | 15 | CE |
| 25 | 27 | F | 14 | CE |
| 26 | 38 | F | 14 | CE |
| 27 | 22 | F | 13 | CE |
| 28 | 37 | M | 13 | CE |
| 29 | 26 | F | 13 | CE |
| 30 | 20 | F | 12 | CE |
