# Supplementary material for: Lateral Crural Mid-Down Flap Technique in Primary Rhinoplasty
Source: Aesthetic Plast Surg. 2025 May 20;49(19):5431–9. doi: 10.1007/s00266-025-04896-8 (PMC12594689; doi:10.1007/s00266-025-04896-8)
Supplement: Supplementary file 2 — Supplementary file2 (DOCX 14 KB) [file 266_2025_4896_MOESM2_ESM.docx]

Supplementary Table 2. Numerical data of the tests used in the study.

| Patient No | Group | SCHNOS Pre-op | SCHNOS Post-op | NPQ Pre-op | NPQ Post-op | VAS Pre-op | VAS Post-op |
| --- | --- | --- | --- | --- | --- | --- | --- |
| 1 | LCMF | 48 | 6 | 30 | 5 | 48 | 90 |
| 2 | LCMF | 48 | 12 | 25 | 5 | 60 | 76 |
| 3 | LCMF | 34 | 0 | 35 | 0 | 40 | 90 |
| 4 | LCMF | 60 | 0 | 50 | 10 | 36 | 90 |
| 5 | LCMF | 72 | 0 | 50 | 15 | 52 | 84 |
| 6 | LCMF | 90 | 2 | 50 | 0 | 36 | 92 |
| 7 | LCMF | 80 | 2 | 35 | 10 | 42 | 84 |
| 8 | LCMF | 72 | 12 | 50 | 15 | 36 | 88 |
| 9 | LCMF | 26 | 4 | 45 | 10 | 30 | 82 |
| 10 | LCMF | 76 | 4 | 30 | 10 | 30 | 88 |
| 11 | LCMF | 84 | 4 | 25 | 20 | 42 | 88 |
| 12 | LCMF | 98 | 8 | 40 | 20 | 28 | 82 |
| 13 | LCMF | 88 | 10 | 20 | 10 | 34 | 84 |
| 14 | LCMF | 56 | 8 | 15 | 30 | 36 | 74 |
| 15 | LCMF | 82 | 6 | 25 | 5 | 44 | 98 |
| 16 | CE | 42 | 20 | 5 | 25 | 32 | 90 |
| 17 | CE | 58 | 0 | 5 | 20 | 44 | 78 |
| 18 | CE | 46 | 0 | 20 | 30 | 52 | 88 |
| 19 | CE | 50 | 8 | 35 | 40 | 48 | 94 |
| 20 | CE | 72 | 18 | 40 | 45 | 36 | 80 |
| 21 | CE | 68 | 10 | 25 | 65 | 42 | 86 |
| 22 | CE | 80 | 20 | 50 | 60 | 34 | 76 |
| 23 | CE | 76 | 18 | 50 | 45 | 24 | 76 |
| 24 | CE | 48 | 10 | 30 | 30 | 56 | 80 |
| 25 | CE | 58 | 78 | 45 | 55 | 46 | 62 |
| 26 | CE | 74 | 8 | 30 | 45 | 44 | 74 |
| 27 | CE | 90 | 16 | 45 | 50 | 40 | 74 |
| 28 | CE | 48 | 2 | 25 | 40 | 38 | 94 |
| 29 | CE | 54 | 2 | 20 | 30 | 30 | 82 |
| 30 | CE | 80 | 2 | 20 | 20 | 42 | 92 |
